# Unforeseen Impact of the IV Fluid Shortage on Patient Safety: A Case of Inadvertent Intra-arterial Injection During General Anesthesia

**DOI:** 10.7759/cureus.80722

**Published:** 2025-03-17

**Authors:** Luke M Johnson, Christopher Evans, Jennifer Bratton, Matthew H Johnson

**Affiliations:** 1 Anesthesiology, Virginia Commonwealth University School of Medicine, Richmond, USA; 2 Anesthesiology, Mountain West Anesthesia, Lehi, USA

**Keywords:** anesthesia complications, continuous iv infusion, hurricane helene, inadvertent catheter placement, intra-arterial injection, iv fluid shortage, safety in anesthesiology

## Abstract

Accidental intra-arterial injection of medications is rare but can have devastating results. There have been many case reports written over the years illustrating this issue and the various sequelae, ranging from minimal impact on the patient to permanent tissue damage and even limb amputation. We report on a surgical case that was performed from start to finish through an unrecognized intra-arterial catheter. This case is unique as it was performed in November 2024 in the United States, which was in the midst of a severe intravenous fluid shortage due to Hurricane Helene. As a result, this case and thousands of others were done without a running IV. Instead, this case was performed by pushing medications through an IV and then flushing in the medications with a small amount of fluid from a syringe in an effort to conserve IV fluids for urgent or emergent use. We postulate that due to not having a running IV, this accidental intra-arterial catheter went unrecognized in a 90-minute surgical case. In all likelihood, with a running IV connected to an intra-arterial catheter, the IV would not have functioned normally and in troubleshooting the IV, the error would have been recognized much sooner. Consequently, the patient would not have been exposed to the dangers of receiving medications through an intra-arterial catheter. This case highlights the critical role of continuous IV fluids in detecting misplaced catheters and underscores the importance of reinstating this practice as soon as supply constraints allow.

## Introduction

Inadvertent intra-arterial (IA) cannulation and medication administration is a rare but well-documented complication in anesthesiology practice. Though perhaps underreported [1], the incidence of unintentional IA cannulations and injections is historically reported to range from 1 in 56,000 to 1 in 3,440 [2]. The potential consequences can be severe, ranging from temporary tissue ischemia to permanent nerve damage or limb loss. The recognition of inadvertent IA cannulation often occurs shortly after beginning continuous fluid administration, due to characteristic signs such as bright red pulsatile blood return or resistance during fluid administration [3].

However, the severe nationwide shortage of intravenous (IV) fluids in late 2024 [4] complicated IA cannulation recognition. Due to the shortage, healthcare facilities were forced to implement strict conservation measures, significantly altering routine anesthesia practices. Many facilities adopted protocols that eliminated the use of maintenance IV fluids during routine surgical cases, instead administering medications through intermittent pushes followed by minimal volume flushes [4]. This departure from the standard practice of maintaining a continuous IV fluid infusion removed an important safety check that reliably assists in identifying misplaced IV lines.

We present a case where an inadvertent IA catheter remained unrecognized during the entire surgical procedure, resulting in the administration of multiple medications through the arterial line. This case uniquely illustrates how the IV fluid shortage created conditions that delayed the recognition of this potentially serious complication. Our experience provides compelling evidence that, in addition to the many safety benefits of running IVs, continuous infusions serve as a crucial safety mechanism for the early detection of misplaced catheters.

## Case presentation

A healthy 23-year-old female (65.8 kg) patient presented to an outpatient surgery center for right anterior cruciate ligament repair after a sports injury. During preoperative preparation, a 22 gauge IV was inadvertently placed in the left brachial artery. The IV catheter had a ‘Blood Control (BC)’ feature so abnormal backflow was not detected at the time of IV placement. The pre-op nurse who placed the IV did not recognize the incorrect placement, flushed a few ml of normal saline through the IV, attached a short small bore extension to the catheter, and taped it in place. The patient was taken to the OR and premedicated with 4 mg of midazolam and 100 mcg of fentanyl which was inadvertently administered intra-arterially. An adductor canal block was performed under ultrasound guidance using 10 cc of 0.5% bupivacaine and 10 cc of Exparel. General anesthesia was then induced with a 200 mg bolus of propofol. The patient complained of burning in the arm with the propofol bolus, but her reaction was not deemed significantly different from many patients who experience discomfort with injection of propofol. Other medications received during the case included: fentanyl 100 mcg (additional), cefazolin 2 g, dexamethasone 10 mg, ondansetron 4 mg, hydromorphone 1 mg, and a propofol infusion running at 100 mcg/kg/min (64 minutes for a total of 421 mg). As the case occurred during a severe fluid shortage, the medications were pushed and then flushed using a small amount of normal saline in prefilled syringes.

At the end of the 90-minute case, when the drapes were pulled down, the anesthesiologist noted that the patient’s left dorsal hand and palm seemed more erythematous and edematous than the right. Concerned, the anesthesiologist drew blood without a tourniquet and blood drew easily back with little resistance. They then placed a normal saline syringe on the hub of the supposed IV catheter, which drew back bright red blood and visualized pulsatility, thus confirming the IV to be intra-arterial. The intra-arterial catheter was immediately removed, pressure was held, and a dressing was placed. A new 20 gauge IV was placed in the contralateral arm.

The patient was taken to the post-anesthesia care unit (PACU) where anesthesia emergence was uneventful. Initially, the patient had no pain in her left hand, though it was still erythematous and edematous. The sensation of the left hand was intact and capillary refill was <3 seconds on the hands bilaterally. Over the next 30 min to 60 min, the swelling reduced and the sensation remained intact. Notably, during the first hour in PACU, the patient developed several non-tender, flat, violaceous lesions on her left forearm. Over the next hour, the lesions regressed slightly, sensation remained intact in her left upper extremity, and no new pain developed. The swelling and erythema continued to decrease. 

After discussing the potentially devastating sequelae of intra-arterial medication injection with the surgery center medical director and a vascular surgeon, the patient was transferred to a nearby in-patient full-service hospital for further observation and to have ready access to more aggressive treatment if needed. The patient was admitted to the hospital under the care of the hospitalist service with vascular surgery consulting. Frequent checks and examinations of the affected arm were performed throughout the evening and overnight. No apparent complications or worsening symptoms were found during her observation in the hospital. The patient was discharged home around noon the next day with careful instructions on things to watch for and triggers to return to the hospital. Ultimately, without any therapeutic intervention, the erythema, edema, and violaceous lesions resolved and the patient did not suffer any neurological insults, tissue damage, or loss of function.

## Discussion

This case highlights the unintended consequences of resource conservation measures implemented during the United States 2024-2025 intravenous fluid shortage. This fluid shortage was caused by supply chain interruptions resulting from the devastating damage caused by Hurricane Helene [4]. While inadvertent IA cannulation is a known complication, its delayed recognition in this case directly stemmed from the absence of a continuous fluid infusion that would typically help reveal arterial placement through increased resistance or backflow.

The medications administered in this case included midazolam, propofol, fentanyl, cefazolin, dexamethasone, ondansetron, and hydromorphone. The literature documents significant variations in tissue toxicity among different medications when administered intra-arterially. Higher lipid-solubility and osmolality of medications are associated with more severe sequelae [3]. For example, diazepam, a highly lipid-soluble agent, has been associated with amputation when injected intra-arterially in multiple reports [5-7]. Lokoff and Maynes created a table documenting over 30 instances of accidental intra-arterial injection of medication and reported the various outcomes ranging from no deficits to amputation, necrosis, and compartment syndrome [1]. Our patient experienced transient effects including erythema, edema, and non-tender violaceous lesions, all of which resolved without long-term complications. A tissue ischemia score, based on color, capillary refill, sensory function, and temperature, can help predict extremity outcomes in cases of intra-arterial drug injection [8]. Scores ≤2 are associated with better outcomes [8]. Given our patient’s mild presentation and resolution of symptoms, their tissue ischemia score likely fell within this favorable range, aligning with the documented positive prognosis [8].

This case also demonstrates the importance of prompt recognition and management of suspected IA cannulation. The anesthesiologist's observation of changes in the patient's hand, followed by immediate confirmation and intervention, likely contributed to preventing more serious complications. The decision to transfer for further observation allowed for monitoring of any delayed manifestations of vascular injury.

## Conclusions

While the IV fluid shortage of 2024-2025 necessitated adaptations in standard anesthesia practice, this case highlights an unintended patient safety risk associated with eliminating continuous IV fluid infusions. The incident underscores that, in addition to providing physiological support, running IV fluids serve as a crucial safety mechanism for the early recognition of misplaced catheters.

Given the potential for severe complications from unrecognized intra-arterial cannulation, we strongly advocate for the immediate reinstatement of continuous IV fluid infusions as soon as supply constraints allow. Furthermore, this case serves as a cautionary example for future resource-conservation efforts, reinforcing the need to balance conservation measures with patient safety.

## References

[1] Lokoff A, Maynes JT (2019). The incidence, significance, and management of accidental intra-arterial injection: a narrative review. Can J Anaesth.

[2] CO SM (1948). Accidental intra-arterial injection of drugs. Lancet.

[3] Sen S, Chini EN, Brown MJ (2005). Complications after unintentional intra-arterial injection of drugs: risks, outcomes, and management strategies. Mayo Clin Proc.

[4] (2025). Anesthesia Patient Safety Foundation. The sterile intravenous (IV) fluid shortage crisis update. https://www.apsf.org/article/the-sterile-intravenous-iv-fluid-shortage-crisis-update/.

[5] Iblher N, Stark GB, Penna V (2011). Necrosis of the 4th and 5th digits after intra-articular injection of diazepam into the wrist. Case Rep Surg.

[6] Mirzatolooei F, Afshar A (2008). Intravenous injection of diazepam to cubital vein can be complicated by accidental intra-arterial penetration and gangrene. Arch Iran Med.

[7] Joist A, Tibesku CO, Neuber M, Frerichmann U, Joosten U (1999). [Gangrene of the fingers caused by accidental intra-arterial injection of diazepam]. Dtsch Med Wochenschr.

[8] Treiman GS, Yellin AE, Weaver FA, Barlow WE, Treiman RL, Gaspar MR (1990). An effective treatment protocol for intraarterial drug injection. J Vasc Surg.

